# Piperazidine

**Published:** 1891-05-02

**Authors:** 


### THERAPEUTICS.

### PIPERAZIDINE.

This base and its hydrochlorite was brought under the notice of the meeting of the Therapeutical Society of Paris on the 28th of January. It is entirely new to most English practitioners, but it is a drug that promises to be of service in lithiasis. It has already been administered as a medicine both in Germany and in Russia. M. Bardet states that piperazidine $C_2\ Az_2\ H_{10}$ is allied to piperidine. Attempts have been made to demonstrate its chemical identity with the spermine of Scheiner, to which, as a matter of fact, it bears a resemblance; but M. Bardet has never observed in its action the stimulating properties attributed to the latter substance. The urate of piperazidine is soluble in forty-seven times its weight of water—that is to say, the urate is very much more soluble than the urate of lithium, which requires three hundred and forty times its weight of water to dissolve it. This fact had encouraged M. Bardet to administer piperazidine in gout. The trials that he made on three patients had proved that one can easily inject, without setting up any degree of irritation, as much as forty centigrammes of the hydrochlorite of piperazidine in the neighbourhood of the affected joints. In the case of one patient, an old sufferer from gravel, he had induced, by doses of thirty centigrammes daily, a diminution and even an abolition of deposits, together with an increase in the amount of soluble urates. At the same time, M. Bardet states that he quotes these facts merely as an instance of its action, and not to form the basis of premature conclusions. This action of piperazidine in dissolving uric acid was of greater importance, to his mind, than its stimulating properties. M. Vogt had also made use of piperazidine, but, contrary to the experience of M. Bardet, he had observed a diminution in the amount of soluble urates and an increase in the urea, and stated that according to the chemist who had prepared the substance this could be explained by the oxidation of the urate formed. M. Bardet had not noticed any stimulating effect from taking piperazidine, and, as M. Boymond remarked, this general absence of stimulation confirmed the opinion of several chemists who refuse to consider piperazidine as analogous to spermine. Ebstein and Sprague, in the *Berlin. Klin. Woch.*, report a case treated with piperazidine. The patient was thirty years of age, and dated his troubles from exposure on a cold, damp night nine years ago, setting up some bladder inflammation. He had attacks at intervals, till he was admitted to the hospital wards. He then complained of pain in the glands and in the groin, on micturition. The urine contained uric acid crystals and was faintly acid; no albumen. Piperazidine was administered in daily doses of from fifteen to thirty grains simply dissolved in water, and were well borne by the patient. A careful analysis of the urine was made nearly every day, giving the amount of uric acid and of urea (as well as other facts of importance) passed in the preceding twenty-four hours. Their observations did not confirm the reports that by the administration of piperazine the total quantity of uric acid is reduced to one-third, and that an excess of urea is excreted. This remedy, possessing in so eminent a degree the property of dissolving uric acid in the form of urates, is certainly well worth a careful investigation.

